# Nickel Vanadium Oxyphosphide Nanosheets with Synergistic Metal–Phosphide Interfaces for Fast and Durable Lithium Storage

**DOI:** 10.1021/acsaem.5c01817

**Published:** 2025-09-02

**Authors:** Vivek Kumar Singh, Idan Bar-lev, Keren Shwartsman, Srijith, Debabrata Mandal, Munseok S. Chae, Jeffrey D. Henderson, Mark C. Biesinger, Bibhudatta Malik, Gilbert Daniel Nessim, Daniel Sharon

**Affiliations:** † Department of Chemistry, Center for Nanoscience and Nanotechnology, 26742 Hebrew University of Jerusalem, Jerusalem 9190401, Israel; ‡ Department of Chemistry, Bar Ilan Institute for Nanotechnology and Advanced Materials, 26731 Bar Ilan University, Ramat Gan 5290002, Israel; § Department of Nanotechnology Engineering, 34998 Pukyong National University, Busan 48547, Republic of Korea; ∥ Surface Science Western, 6221 The University of Western Ontario, London, Ontario N6G 0J3, Canada; ⊥ Department of Chemistry, The University of Western Ontario, London, Ontario N6A 5B7, Canada

**Keywords:** lithium-ion batteries (LIBs), nickel phosphide (Ni_2_P), layered double hydroxide (LDH), nickel vanadium oxyphosphide (NVOP), nanosheets

## Abstract

Achieving high capacity, long-term stability, and fast charge–discharge capability remains a central challenge in the development of advanced anode materials for lithium-ion batteries. In this work, we present nickel vanadium oxyphosphide (NVOP) nanosheets synthesized via controlled thermal phosphorization of NiV-layered double hydroxide (NiV-LDH). The resulting multiphase structure, composed of conductive Ni_2_P and redox-active vanadium oxides, delivers an initial discharge capacity of 1345 mAh/g and retains 442 mAh/g after 200 cycles at 0.1 A/g, with Coulombic efficiency stabilizing near 99.5%. NVOP also demonstrates excellent rate performance, maintaining 359 mAh/g at a high current density of 1.0 A/g. Electrochemical and structural characterization suggest that the improved cycling stability and rate capability may stem from the multiphase architecture, which integrates conductive and redox-active components within a porous nanosheet framework. These findings underscore the potential of direct phosphorization of mixed-metal layered hydroxide precursors as an effective strategy for constructing high-performance, durable anode materials for next-generation lithium-ion batteries.

## Graphical abstract

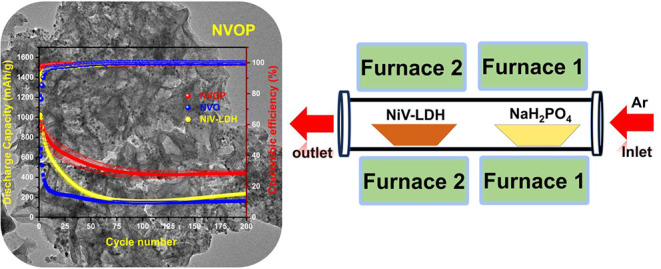

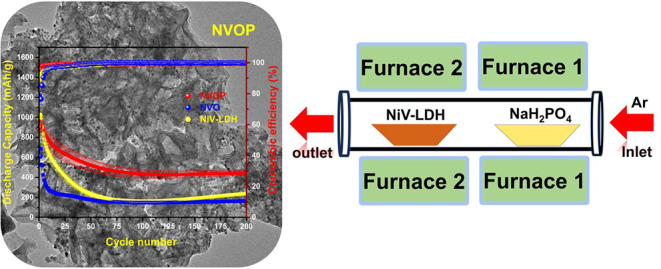

## 1 Introduction

Lithium-ion batteries (LIBs) have become the dominant choice for energy storage technology for applications such as electric vehicles due to their long cycling life and high energy density.^1–3^ However, further improvements in LIBs performance are needed to meet the growing demands for higher energy densities. The conventional graphite anode (theoretical capacity 372 mAh/g) is reaching its limits, spurring extensive research into alternative anode materials.^4,5^

Transition-metal oxides and phosphides have emerged as promising anode candidates for next-generation LIBs, offering high theoretical capacities, good chemical and thermal stability, and low operating potentials.^4,6–14^ Among them, metal-rich phosphides such as nickel phosphide (Ni_*x*_P_*y*_) are particularly attractive due to their high theoretical capacity (542 mAh/g), cost-effectiveness, and environmental friendliness.^15,16^ Despite these advantages, the practical application of pure nickel phosphide anodes, especially Ni_2_P, remains limited by several challenges. These include low specific surface area, poor electronic conductivity, large volume expansion during charge and discharge, a tendency to agglomerate, and slow Li^+^ ions diffusion.^17–20^ The large volume expansion causes severe pulverization and detachment of the active material, while the poor conductivity and slow diffusion impair redox kinetics and diminish both capacity utilization and retention.^21^

Recent efforts to improve the cyclability of Ni_*x*_P_*y*_ electrodes have focused on nanoscale morphology engineering and carbon composite integration. For example, Lu et al. prepared porous Ni_2_P nanosheets, which demonstrated a reversible discharge capacity of 379.8 mAh/g for 50 cycles within an operational potential window of 0.1–3 V (vs Li/Li^+^) at a current density of 0.1 A/g.^22^ The improved rate capability was attributed to the 2D structure, which shortened Li-ion diffusion paths and buffered mechanical stress during cycling. Cai et al. developed a rose-like three-dimensional (3D) hierarchical structure, achieved through the self-assembly of two-dimensional (2D) Ni_2_P nanoflakes on reduced graphene oxide (rGO). This novel configuration exhibited a discharge capacity of 330.5 mAh/g after 100 cycles at a current density of 0.1 A/g.^23^ The exceptional electrochemical performance can be ascribed to the synergistic interaction between the three-dimensional hierarchical rose-like Ni_2_P and reduced graphene oxide (rGO). In this configuration, rGO functions as a conductive framework for Ni_2_P and alleviates structural degradation during prolonged cycling.

In addition to morphology and carbon composite design, another widely explored strategy involves the construction of heterostructures that combine Ni_2_P with additional lithium-active phases.^24–28^ For example, Yu et al. developed a carbon-confined V_2_O_3_/Ni_2_P/C heterostructure as a lithium-ion battery anode, which exhibited improved cycling stability of 440 mAh/g after 200 cycles.^28^ Density functional theory (DFT) calculations attributed the enhanced performance to strong covalent bonding between Ni_2_P and V_2_O_3_, which facilitated charge transfer and reinforced structural integrity compared with the individual components. Despite the improved performance achieved through carbon-based composites and heterostructure formation, most of these approaches rely on physical mixing or postsynthetic assembly, which often result in limited phase integration and weak interfacial connectivity. These limitations restrict the ability to fully exploit the synergistic potential of multicomponent systems.

One approach that can help overcome the limitations associated with physical mixing and poor phase integration in Ni_2_P-based composites is the in situ formation of a multiphase heterostructure. In this work, we introduce a nickel vanadium oxyphosphide (NVOP) material composed of interconnected Ni_2_P, NiO, and V_2_O_3_ phases, synthesized through chemical vapor deposition (CVD) phosphorization of a nickel–vanadium-layered double hydroxide (NiV-LDH) precursor. This method enables simultaneous phase formation and structural templating, resulting in a well-integrated two-dimensional nanosheet framework with coherent interfaces and uniform elemental distribution. The incorporation of oxygen and vanadium is expected to enhance electronic conductivity and to introduce additional redox-active sites, thus enabling multielectron reactions for higher capacity.^29–31^ Furthermore, the hybrid Ni–V–P and oxide phases synergistically improve Li^+^ diffusion kinetics, while the nanosheet morphology provides a large surface area and short ion diffusion paths, boosting rate capability.^24^

While multiphase Ni_2_P-based composites have been studied, the NVOP system developed here features a unique combination of phases, integrated through a synthesis strategy that promotes strong interfacial coherence and structural uniformity. The NVOP anode demonstrates outstanding electrochemical performance with high capacity, stable cycling, and rapid charge–discharge capabilities, compared to its individual components. This performance arises from the synergistic interaction of the multiphase composition and the advantages of in situ phase formation within a nanosheet architecture, highlighting this synthetic route as a promising platform for the development of high-performance multiphase LIBs anodes.

## 2 Experimental Section

### 2.1 Material Synthesis

Synthesis of nickel vanadium-layered double hydroxide (NiV-LDH): The nickel vanadium (NiV-LDH) was synthesized through a one-pot hydrothermal method. In this typical procedure, 0.6 mM nickel nitrate hexahydrate (Ni­(NO_3_)_2_·6H_2_O, 98%, Sigma-Aldrich), 0.5 mM vanadium­(III) chloride (VCl_3_, 98%, Alfa Aesar), 4 mM (240 mg) urea (CH_4_N_2_O, 98%, Sigma-Aldrich), and 6 mM (220 mg) ammonium fluoride (NH_4_F, 99.9%, Thermo Scientific) were mixed into 30 mL of deionized water (DI) and stirred for 30 min to form a uniform solution. Subsequently, the solution was transferred to a 50 mL Teflon-lined stainless-steel autoclave, where it was maintained at a temperature of 130 °C for a period of 12 h. Finally, the resulting sample was rinsed several times with DI water and ethanol and then dried for 12 h at 60 °C. The final product was referred to as NiV-LDH.

Synthesis of nickel vanadium oxyphosphide (NVOP) and nickel vanadium-layered oxide (NVO): The prepared NiV-LDH and 500 mg of sodium dihydrogen phosphate (NaH_2_PO_4_, 99%, Sigma-Aldrich) were placed at separate locations within a two-tube furnace in series (using the same quartz tube), with NaH_2_PO_4_ positioned in the upstream furnace and NiV-LDH positioned in the downstream furnace. The phosphorization process was conducted in an argon environment to prevent oxidation. The temperature was then raised to 350 °C at a rate of 2 °C per minute and maintained at 350 °C for 2 h. Once it naturally cooled to room temperature, NVOP was obtained. Nickel vanadium oxide (NVO) was synthesized through a similar process but without the inclusion of sodium dihydrogen phosphate (NaH_2_PO_4_, 99%, Sigma-Aldrich).

#### 2.1.1 Material Characterization

The synthesized samples were characterized using powder X-ray diffraction (PXRD) with a Bruker D8 (Billerica, MA) under conditions of 40 kV and 44 mA, utilizing Cu Kα radiation (λ = 1.5418 Å). Rietveld refinements for each sample were conducted using powder profile refinement software GSAS1,^32^ with initial structural models sourced from the Materials Project.^33^ The morphology and structure of all samples were examined by using a high-resolution scanning electron microscope (HR-SEM, FEI, Magellan 400L, Hillsboro, OR) that included an energy-dispersive spectrometer (EDS). This analysis was further complemented by transmission electron microscopy (TEM) (JEOL JEM-2100, Tokyo, Japan). The X-ray photoelectron spectroscopy (XPS) analysis was conducted by using a Thermo Scientific Nexsa spectrometer. This instrument is equipped with a monochromated, microfocused, low-power Al Kα X-ray source, which functions at an energy of 1486.7 eV. Survey spectra were collected using a pass energy of 200 eV and a step size of 1 eV, while high-resolution spectra were collected using a pass energy of 20 eV and a step size of 0.1 eV. For XPS analyses, all samples were mounted in electrical isolation to the stage to avoid differential charging. All spectra were collected with the instrument charge neutralization function and referenced to adventitious carbon (284.8 eV).^34,35^ Fitting routines were based on previously published data and procedures.^36–38^ For oxidized forms of Ni, which show complex multiplet splitting behavior,^36^ previously published peak constraints were used to replicate the envelope of high-quality standards (e.g., NiO, Ni­(OH)_2_, Ni_3_(PO_4_)_2_). These fits were further guided by stoichiometric information available from survey spectra.^38^ Surface area measurements were conducted using a NOVA 3200E Quantachrome (BET, Brunauer–Emmett–Teller).

#### 2.1.2 Electrochemical Characterizations

The working electrodes were fabricated by using a slurry coating method. This slurry consisted of 80 wt % NVOP nanosheets, 10 wt % carbon black, and 10 wt % polyvinylidene fluoride (PVDF) dissolved in *N*-methylpyrrolidinone (NMP). It was applied to a copper foil with a doctor blade, achieving a wet coating thickness of 80 μm. The electrodes were then punched out and dried overnight at 100 °C in vacuum before being placed in an Ar-filled glovebox. The weight of the active material is estimated to be approximately 1.25 to 1.45 mg for each half-cell. For electrochemical investigations, CR2032-type coin cells were assembled inside a glovebox. These cells used metallic lithium foil as the cathode, around 50 μL of an EC: EMC (3:7 by volume) solution with 1 M LiPF_6_ electrolyte (LP57 from BASF) as the electrolyte, and two polypropylene (PP) microporous films as separators. Galvanostatic charge–discharge tests were carried out using the Neware battery program control test system (CT-4008Tn-5 V50 mA-HW B) at a current density of 0.1 A/g, within a voltage range 0.1–3.0 V, and at room temperature (30 °C). Cyclic voltammetry (CV) experiments were performed using a VSP-3e Biologic potentiostat electrochemistry workstation, with a scanning speed of 0.1 mV/s in the voltage range of 0.1 to 3.0 V relative to Li/Li^+^. Electrochemical impedance spectroscopy (EIS) measurements were conducted over a frequency range from 0.01 Hz to 100 kHz.

## 3 Results and Discussion

The step-by-step synthesis of NVOP is illustrated in Figure 1. Initially, NiV-LDH nanosheets were synthesized via a one-pot hydrothermal method. These were then thermally treated in a two-furnace system flowing in a PH_3_ atmosphere, generated in situ by the pyrolysis of NaH_2_PO_4_. During this CVD process, NiV-LDH is simultaneously converted into nickel vanadium oxide and reacts with PH_3_ to incorporate phosphorus, yielding the final NVOP product. For comparison, a control sample was also annealed under identical thermal conditions but without phosphorization, resulting in the formation of NVO instead. The phosphorization temperature was set to 350 °C, based on preliminary screening across a range of temperatures (350–650 °C), where 350 °C provided the optimal balance between phase formation and electrochemical performance (Figure S7).

**Figure 1:**
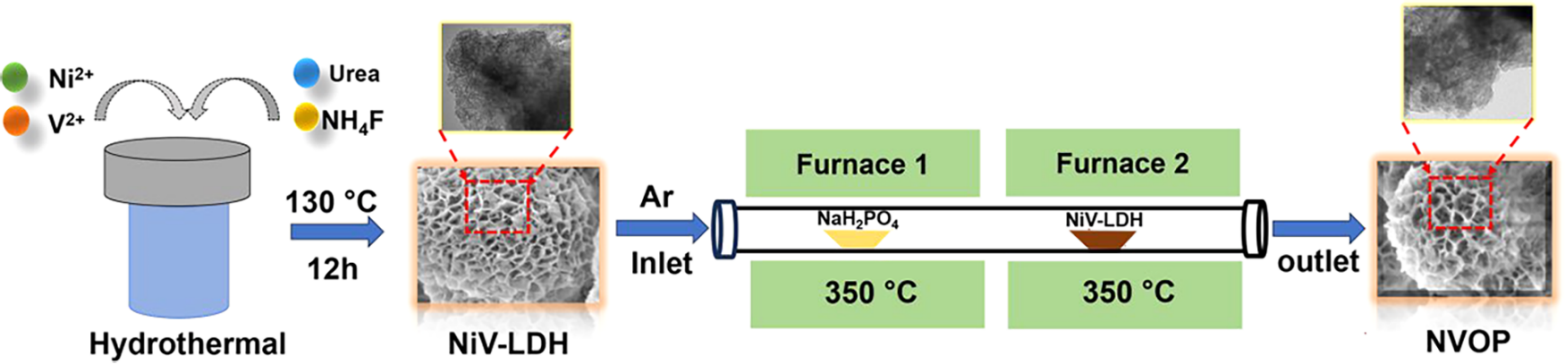
Schematic representation of the step-by-step synthesis procedure of the nickel vanadium oxyphosphide (NVOP).

The structural evolution from NiV-LDH to NVO or NVOP was analyzed by using XRD, as shown in Figure 2a. The XRD pattern of the hydrothermal synthesized NiV-LDH exhibits primary peaks at ∼18.63°, 34.80°, 45.66°, and 60.92° corresponding to the (006), (012), (018), and (113) crystal planes of a layered double hydroxide structure (*JCPDS No. 05–1627*).^39,40^ Thermal treatment at 350 °C without phosphorization led to the formation of the NVO sample (red), which exhibits diffraction peaks at ∼32.38° and 36.04°, corresponding to the (104) and (110) planes of V_2_O_3_ (*JCPDS No. 98–004–5695*), along with peaks at ∼43.59° and 63.98°, assigned to the (002) and (022) planes of NiO (*JCPDS No. 98–000–8167*). The coexistence of NiO and V_2_O_3_ in the NVO sample is further supported by Rietveld refinement, as shown in Figure 2b and Table S1, indicating a composition of 79.3% NiO and 20.7% V_2_O_3_.

**Figure 2:**
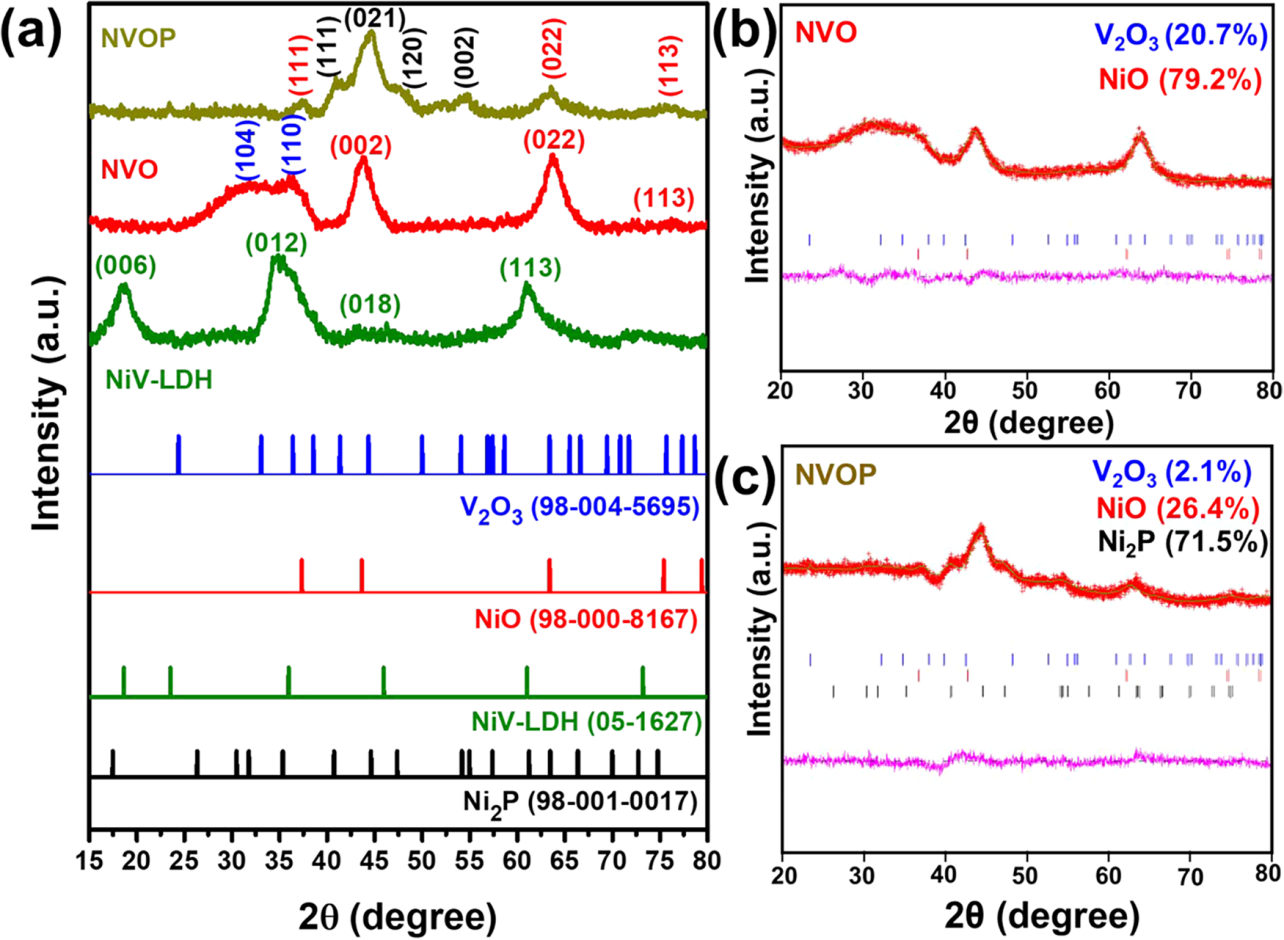
(a) Comparative X-ray diffraction patterns of NiV-LDH, NVO, and NVOP. (b, c) Rietveld refined profile-matching data of NVO and NVOP samples.

After CVD phosphorization, the XRD pattern of NVOP (brown) exhibits diffraction peaks at approximately ∼40.89°, 44.63°, 47.65°, and 54.72°, which correspond to the (111), (021), (120), and (002) planes of hexagonal Ni_2_P (*JCPDS No. 98–001–0017*). In addition, peaks at ∼37.39°, 63.52°, and 75.51° are assigned to the (111), (022), and (012) planes of NiO (*JCPDS No. 98–000–8167*), while residual peaks corresponding to V_2_O_3_ are also observed. The presence of Ni_2_P, NiO, and V_2_O_3_ phases confirms the multiphase nature of the NVOP product and is supported by Rietveld refinement (Figure 2c, Table S2), which quantifies the composition as 71.5% Ni_2_P, 26.4% NiO, and 2.1% V_2_O_3_. This transformation highlights the predominant formation of Ni_2_P during phosphorization while retaining a minor oxide fraction that may influence the structural integrity and electrochemical behavior of the composite.

The morphology and structure of NiV-LDH, NVO, and NVOP were characterized by SEM. Figure 3a,b presents the SEM images of the NiV-LDH, revealing a microsphere-like structure composed of numerous nanosheets oriented perpendicularly to the microsphere surface, with an average microsphere diameter of around ∼2.87 μm. After thermal treatment at 350 °C, the NVO sample (Figure 3c,d) retained its microspherical morphology. Similarly, after thermal phosphorization in PH_3_ gas flow generated from the pyrolysis of NaH_2_PO_4_ in inert gas, the NVOP sample (Figure 3e,f) maintained its structural integrity. The average microsphere diameter of both NVO and NVOP was approximately ∼2.35 μm, indicating that neither thermal treatment nor phosphorization significantly altered the microstructure. EDS elemental mapping (Figure S1) confirms the uniform distribution of Ni, V, P, and O in the NVOP sample, supporting the successful incorporation of phosphorus and the formation of a multiphase structure.

**Figure 3:**
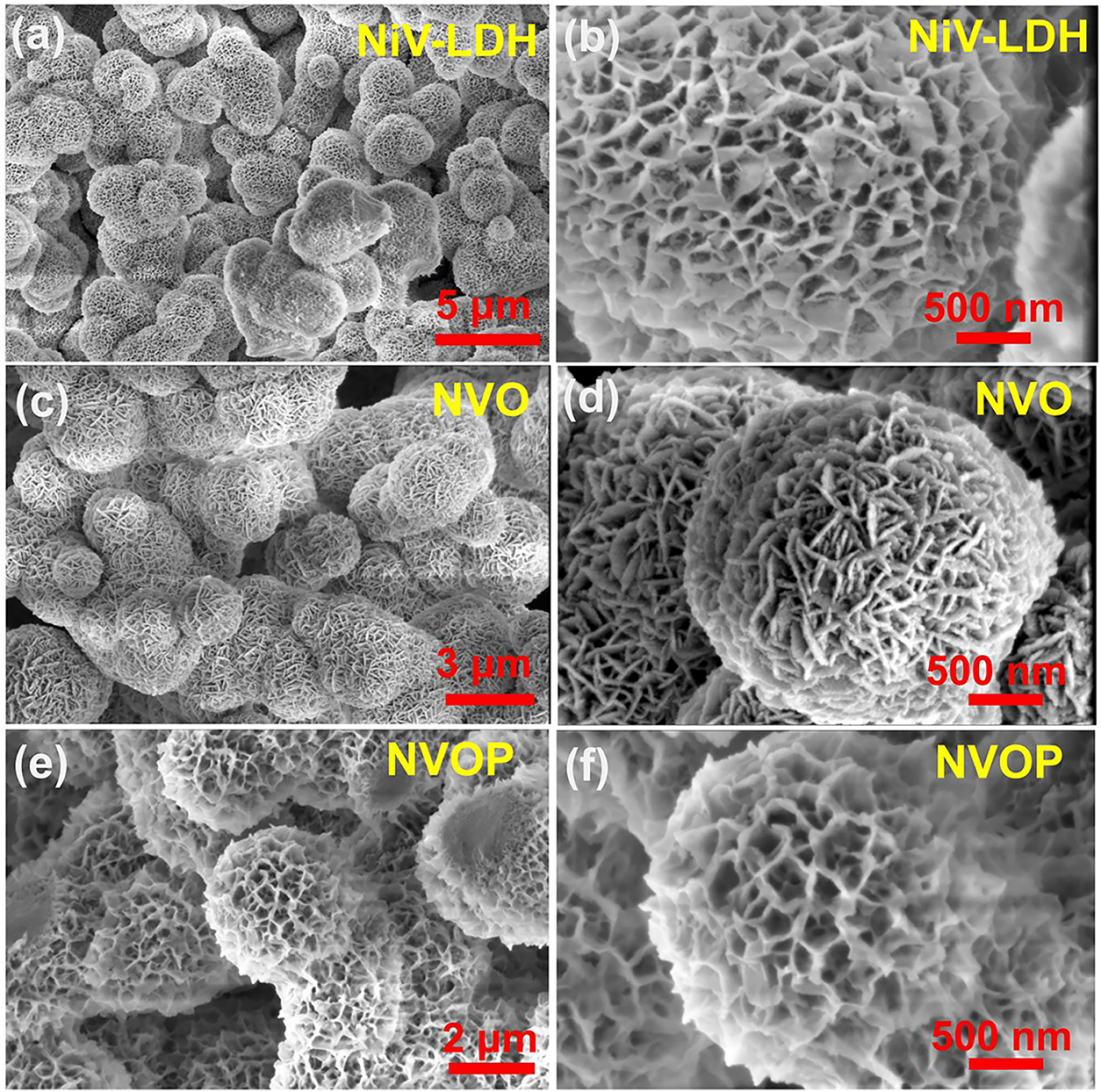
SEM images of (a, b) NiV-LDH, (c, d) NVO, and (e, f) NVOP.

To further investigate the morphology and crystal structure, TEM was performed on the synthesized materials (Figure 4). The TEM image (Figure 4a) shows a nanosheet-like structure, consistent with the SEM observation. The HRTEM image (Figure 4b) exhibits a lattice fringe spacing of 0.214 ± 0.04 nm, corresponding to the (018) crystal plane of NiV-LDH (*JCPDS No. 05–1627*). Figure 4c shows the selected-area electron diffraction (SAED) pattern, displaying characteristic diffraction spots corresponding to the (018) and (113) planes, suggesting the formation of NiV-LHD. The bright diffraction rings in the SAED pattern further indicate that most of the NiV-LHD exhibits a polycrystalline nature, consistent with the XRD data. Figure 4d shows a similar morphology of the NVO after the thermal treatment of the NiV-LDH. The corresponding HRTEM image (Figure 4e) exhibits a lattice fringe of 0.240 ± 0.008 nm, corresponding to either NiO (111) or V_2_O_3_ (110) plane. The SAED pattern (Figure 4f) of NVO exhibits the characteristics of diffraction spots of (002) and (022) for NiO (*JCPDS No. 98–006–9447*) and (312) crystal plane of V_2_O_3_ (*JCPDS No. 98–004–5695*).

**Figure 4:**
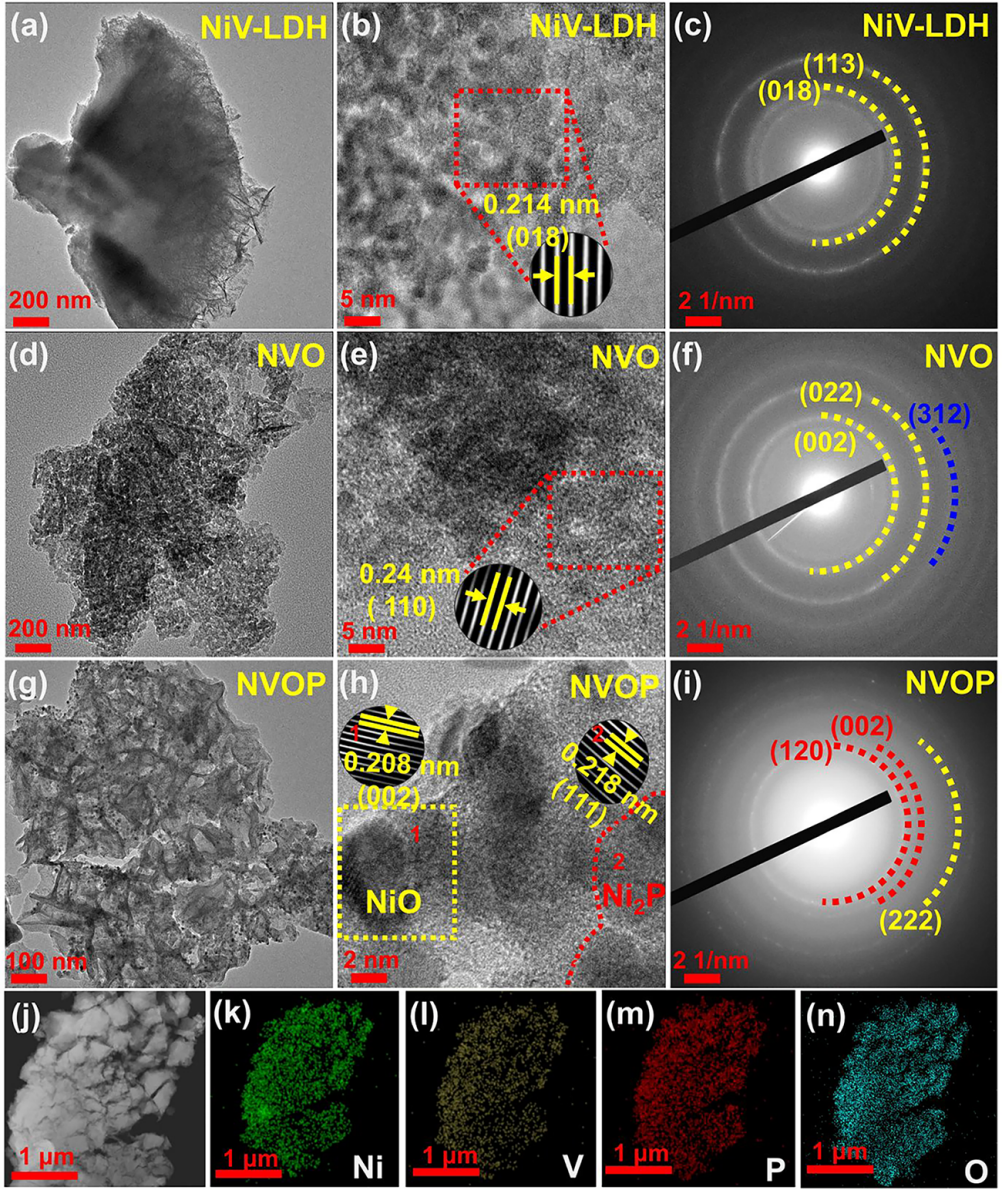
TEM and HRTEM images, along with selected-area electron diffraction (SAED) pattern, of (a–c) NiV-LDH, (d–f) NVO, and (g–i) NVOP, and (j–n) Scanning TEM (STEM) image of NVOP and the EDS mapping of the Ni, V, P, and O elements.

Figure 4g presents the TEM image of the NVOP after the phosphorization of the NiV-LDH, showing that the 2D structure remains intact after the process. The HRTEM images (Figure 4h) show well-defined lattice fringes with the d-spacing of 0.208 ± 0.06 nm (inset 1), corresponding to the (002) crystal plane of NiO (*JCPDS No. 98–000–8167*). Similarly, another region (inset 2) exhibits a d-spacing of 0.218 ± 0.05 nm, corresponding to the (111) crystal plane of Ni_2_P (*JCPDS No. 98–001–0017*). The SAED pattern (Figure 4i) further confirms the phase composition, exhibiting the characteristic diffraction spots of (120), (002), and (222), corresponding to the Ni_2_P and NiO planes, consistent with the XRD data. Figure 4j–n presents dark-field scanning transmission electron microscopy (STEM) images and energy-dispersive X-ray spectroscopy (STEM-EDS) maps of NVOP, demonstrating a uniform distribution of nickel (green), vanadium (yellow), phosphorus (red), and oxygen (cyan) across the NVOP nanosheets.

XPS was performed to analyze the oxidation state and surface chemical composition of the synthesized materials (Figure 5). The XPS survey spectra (Figure S2) of NiV-LDH, NVO, and NVOP show signals consistent with carbon, oxygen, nickel, and small amounts of vanadium. Depending on the sample, small to trace amounts of nitrogen and fluorine were also detected. After the phosphorization process, signals consistent with phosphorus appeared in the NVOP sample, confirming the incorporation of phosphorus, which is in agreement with the EDS mapping.

**Figure 5:**
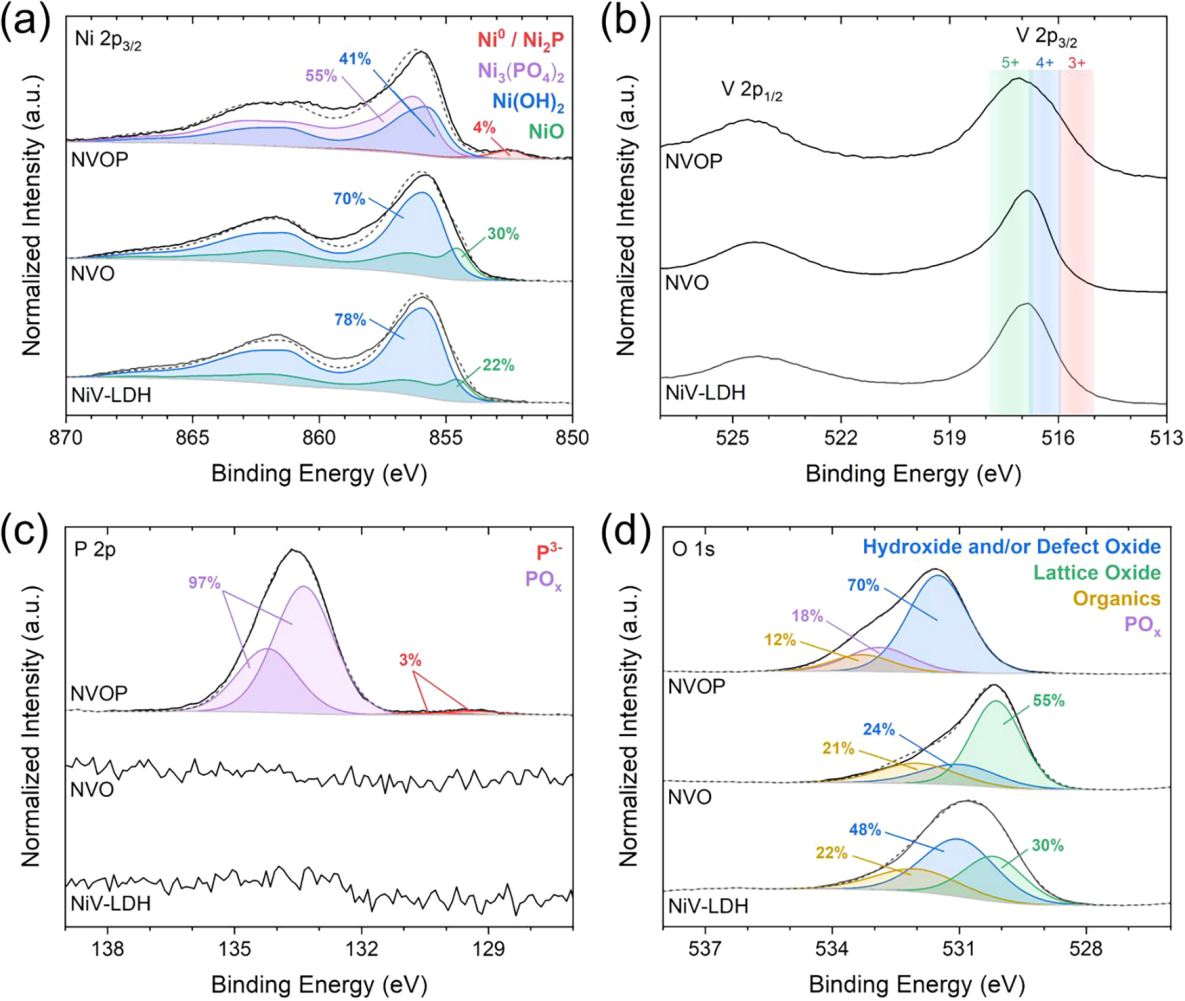
High-resolution spectrum of (a) Ni 2p_3/2_, (b) V 2p, (c) P 2p, and (d) O 1s for NiV-LDH, NVO, and NVOP, illustrating the chemical state evolution upon phosphorization.

Figure 5a presents the high-resolution XPS spectra of Ni 2p_3/2_, revealing the chemical states of nickel in NiV-LDH, NVO, and NVOP. For samples NiV-LDH and NVO, the experimental data were consistent with standard spectra of NiO and Ni­(OH)_2_.^36^ This is consistent with the findings of other researchers.^41–46^ Following phosphorization, a low-binding-energy component was detected in the NVOP samples, corresponding to either nickel phosphide (Ni_2_P) or metallic nickel (Ni^0^). Additionally, adequate fitting required the standard spectra of nickel phosphate (Ni_3_(PO_4_)_2_) on the surface of NVOP samples.^47–51^ As shown in the top spectrum of Figure 5a, XPS peak fitting reveals a surface composition for NVOP dominated by Ni­(OH)_2_ and (Ni_3_(PO_4_)_2_).^52,53^ This highlights the prevalence of phosphate and hydroxide species at the surface, likely due to surface oxidation and phosphate formation during or after phosphorization, while the underlying Ni_2_P phase is more representative of the bulk, as confirmed by XRD.

Figure 5b presents the V 2p XPS spectra, showing a broad signal between ∼514.9 and 517.9 eV corresponding to the V 2p_3/2_ spin–orbit component. The broad envelope observed in this region is unlike that seen in standard reference spectra,^36^ showing very broad signals. For reference, the approximate binding energy ranges of V^3+^, V^4+^, and V^5+^ oxides are indicated above the experimental data. In general, the experimental data extend mainly across the V^4+^ and V^5+^ states, suggesting that V^3+^ is partially oxidized to V^4+^ and V^5+^ during the hydrothermal and CVD processes, consistent with previous reports.^54–57^ The peak observed from ∼522 to 526 eV corresponds to the V 2p_1/2_ spin–orbit component of the V 2p signal.

Figure 5c presents the high-resolution P 2p spectra, where distinct peaks appear exclusively in the NVOP sample, as expected. Peaks at ∼133.4 (2p_3/2_) and ∼134.2 eV (2p_1/2_) are attributed to the oxidized phosphorus (PO_*x*_) species, while additional peaks at ∼129.4 (2p_3/2_) and 130.3 eV (2p_1/2_) correspond to phosphide (P^3–^) species.^58–60^ Notably, for the NVOP sample, the observations made in the P 2p spectra were consistent with those made in the Ni 2p_3/2_ spectra (discussed above). Figure 5d presents the O 1s spectra, where all samples present peaks at ∼530.0, 531.0, and 532.0 eV, which are assigned to lattice oxygen, hydroxide/defect oxide, and organic species, respectively.^41,43^ Notably, a signal consistent with phosphorus–oxygen (PO*_x_*) was detected only in the NVOP sample at ∼532.9 eV.^61^ Since a large degree of overlap exists in this region, stoichiometry was used to guide its contribution to the overall envelope.

The chemical characterization, including XPS, TEM-EDS, and XRD analyses, confirms the successful incorporation of nickel, vanadium, phosphorus, and oxygen into the NVOP framework, leading to a well-defined, multiphase structure. The rational design of NVOP, nickel, contributed to electronic conductivity and electrochemical activity, while vanadium enhances structural integrity and capacity by accommodating multiple oxidation states. Oxygen and phosphorus further improved structural stability, oxygen reinforces mechanical rigidity, whereas phosphorus increases theoretical lithium storage capacity and promotes the formation of a robust solid electrolyte interface (SEI).^62,63^

The surface area and porosity of NiV-LDH, NVO, and NVOP were measured using a BET analysis (Figure S3). The BET-specific surface areas of NVOP, NVO, and NiV-LDH were approximately 58, 22, and 25 m^2^/g, respectively. The pore size distribution analysis confirms the microporous nature of the materials, with average pore diameters of ∼1.42 nm for NVOP, ∼1.65 nm for NVO, and ∼1.58 nm for NiV-LDH samples, as shown in Figure S3b–d. The increase in surface area observed during the phosphorization of NiV-LDH to NVOP is due to the development of a porous structure and the transformation of NiV-LDH into smaller, nanosized particles at higher temperatures. The interaction with NaH_2_PO_4_ results in the creation of more intricate hierarchical nanostructures, which increases surface roughness and increases the total surface area. The increased surface area may provide a greater number of electroactive sites, facilitate faster faradaic reactions, and contribute to enhanced charge storage. Additionally, the porous structure likely promotes efficient electrolyte infiltration and short ion diffusion pathways, which may enhance the rate capability. However, the larger surface area may also lead to more extensive SEI formation due to increased interfacial reactivity, potentially contributing to the pronounced initial irreversible capacity loss observed across all samples. Nevertheless, the improved structural accessibility and morphological stability of NVOP might support sustained cycling performance and a high-rate operation.

The electrochemical behavior of NVOP was evaluated by cyclic voltammetry in Li half-cells at a scan rate of 0.1 mV/s within 0.1–3.0 V (Figure 6a). A broad peak centered around ∼1.1 V appears in the first cathodic scan and fades in subsequent cycles, indicating that electrolyte decomposition and SEI formation primarily occur during the initial lithiation. The second cathodic peak, emerging near ∼0.64 V, is attributed to the conversion of Ni_2_P into metallic Ni and Li_*x*_P phases, along with partial reduction of vanadium to lower oxidation states. This peak stabilizes in subsequent cycles and may also involve additional conversion reactions, including the formation of Li_2_O from residual oxide phases present in the multiphase structure of NVOP.^28,64,65^ Additional cathodic reaction at lower potentials may be associated with lithium storage at defect-rich or interfacial sites within the phosphide and oxide domains of NVOP, consistent with surface-dominated processes observed in similar Ni- and V-based conversion materials.^66^ On the anodic sweep, two distinct oxidation peaks are observed at approximately 1.45  and 2.1 V. These are attributed to the reoxidation of metallic Ni and Li_*x*_P back to nickel phosphide phases, as well as the stepwise oxidation of reduced vanadium species toward higher oxidation states.

**Figure 6:**
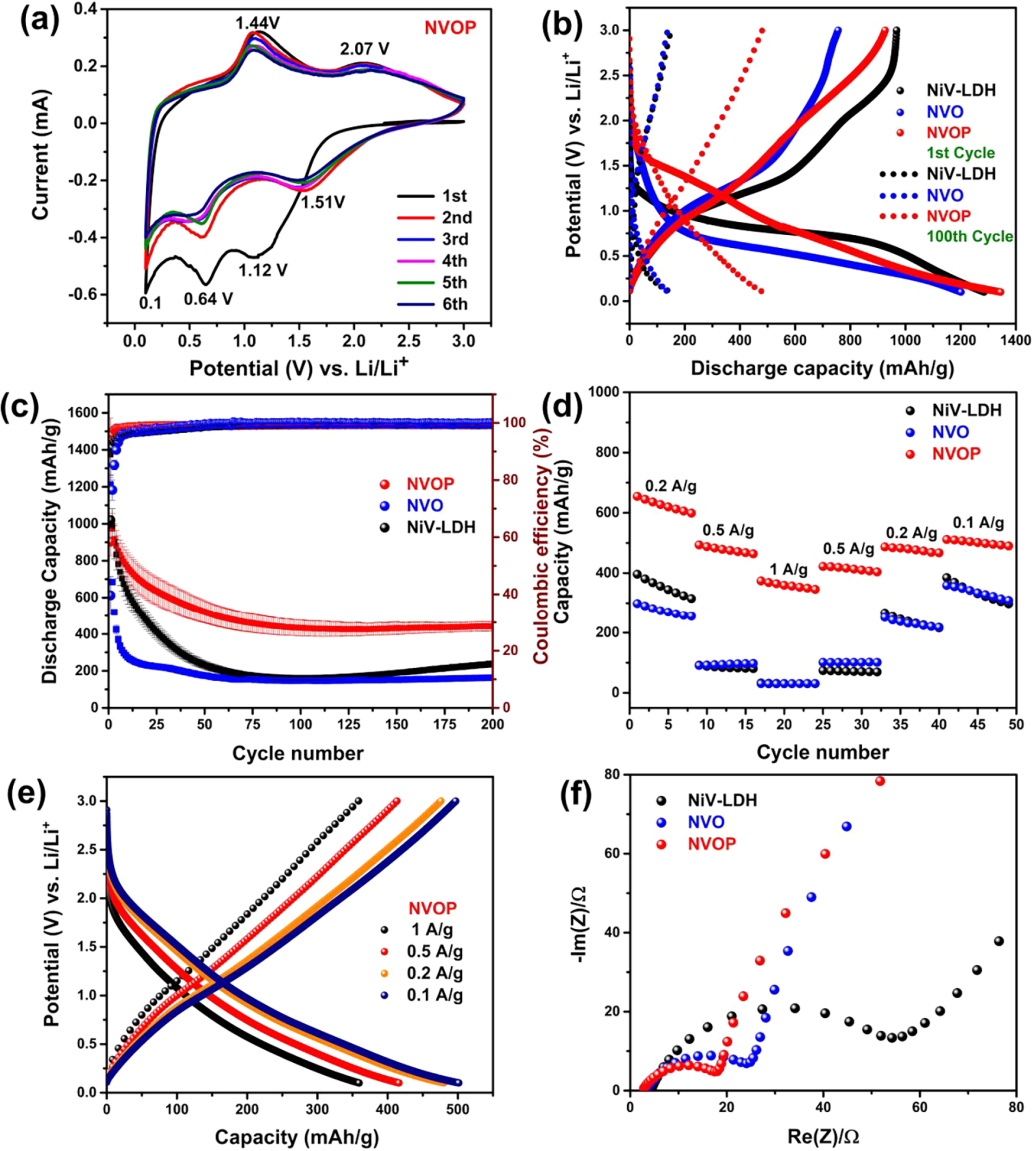
(a) CV curve of NVOP at a scan rate of 0.1 mV/s from 0.1 to 3 V, (b) charge–discharge curves of NVOP, NVO, and NiV-LDH after the initial cycle and hundred cycle at a current density of 0.1 A/g, (c) cycling performance of all electrodes and their corresponding Coulombic efficiency at 0.1 A/g, (d) rate capacity plot of NiV-LDH, NVO, and NVOP electrode at different current density at 0.2, 0.5, and 1 A/g, (e) charge–discharge curves at of NVOP electrode at different current density, and (f) Nyquist plots of NiV-LDH, NVO, and NVOP electrodes.

In contrast to NVOP, the CV curves of NiV-LDH and NVO (Figure S4) exhibit low-intensity and less defined redox behavior. NiV-LDH shows a broad cathodic response centered around 0.3–0.4 V in the first cycle, which becomes less distinct upon cycling. The anodic current is minimal, indicating a limited reversibility. NVO displays an even flatter cathodic signal below 0.3 V with a negligible anodic response, consistent with largely irreversible redox processes. In later cycles, both NiV-LDH and NVO exhibit fading cathodic signals and minimal anodic evolution, indicating poor redox reversibility and limited electrochemical stability. In contrast, NVOP demonstrates stable and well-defined redox peaks over multiple cycles, reflecting a more consistent utilization of its active phases.

The comparative CV analysis suggests that the reversible redox peak at ∼0.64 V in NVOP, absent in NVO and NiV-LDH, is likely associated with Ni_2_P conversion reactions, indicating its dominant role in the capacity contribution. Broader cathodic features below ∼0.5 V, more pronounced in NVOP than in the precursors, may reflect vanadium redox activity contributing to pseudocapacitive behavior. While NiO does not exhibit clear redox features, it may support structural stability within the composite. The higher and more stable current response in NVOP compared to its individual components further implies improved kinetics, potentially due to the conductive Ni_2_P phase and enhanced interfacial integration. These phase-specific roles are consistent with the electrochemical trends but remain hypotheses based on comparative data.

Galvanostatic charge–discharge cycling further demonstrates the superior cycling stability of the NVOP anode material. Figure 6b presents the 1st and 100th charge–discharge voltage curves of the NVOP electrode exhibit a charge/discharge capacity of 962/1345 mAh/g, while the NVO and NiV electrodes show 756/1201 and 967/1284 mAh/g, respectively. The discharge profile of NVOP shows a plateau between 1.0 and 1.4 V, corresponding to SEI formation and initial Li^+^ insertion into V-based oxides and partial reduction of surface Ni/V oxides. Below 1.0 V, a sloping region reflects conversion reactions involving Ni_2_P and Ni/V-based oxide phases, which contribute to the high capacity but also lead to structural changes and irreversible losses during the first cycle; similar features are also observed in the NVO and NiV-LDH electrodes. The reproducibility of these results is further supported by voltage profiles from three independent cells for each anode material, as shown in Figure S5. As cycling progresses, the voltage profiles of NVOP gradually stabilize, following initial structural rearrangements and capacity activation over the first few cycles. After 100 cycles, the electrode retains a high discharge capacity of 484 mAh/g, significantly outperforming NVO (138 mAh/g) and NiV-LDH (150 mAh/g).

The cycling performance and Coulombic efficiency (CE) of all electrodes were further evaluated at 0.1 A/g (Figure 6c). NVOP exhibits an initial discharge capacity of 1345 mAh/g with a CE of 58.39%, indicative of significant irreversible capacity loss during the first cycle. We propose that this loss is primarily due to electrolyte decomposition and solid electrolyte interphase (SEI) formation, as suggested by the broad reduction feature around ∼1.1 V in the initial cathodic scan (Figure 6a), which fades in subsequent cycles. Additional irreversible loss may arise from partial irreversibility in the conversion reactions of Ni_2_P and V_2_O_3_, which can lead to the formation of inactive or poorly reoxidized phases. With continued cycling, the CE increases progressively and stabilizes around 99.46%, indicating highly reversible charge–discharge behavior.^67^ After 200 cycles, NVOP maintains a stable discharge capacity of 441.7 mAh/g, outperforming NiV-LDH (238.1 mAh/g) and NVO (163.9 mAh/g). To assess whether the irreversible capacity is accompanied by structural degradation of the active phases, we conducted ex situ XRD analysis for the NVOP electrode after 100 cycles. The characteristic peaks of Ni_2_P and NiO remain clearly visible (Figure S6), indicating that the multiphase structure is preserved and that the initial capacity loss stems primarily from interfacial reactions rather than permanent phase decomposition.

The rate capability of the electrodes was assessed by varying the current density from 0.2, 0.5, and 1.0 A/g, as shown in Figure 6d. NVOP consistently delivers the highest capacities, with 480, 416, and 359 mAh/g at 0.2, 0.5, and 1.0 A/g, respectively, substantially outperforming NiV-LDH and NVO under identical conditions. This advantage is particularly pronounced at high current densities, where NVOP retains nearly 74.79% of its initial capacity at 1 A/g, compared to significant drops in the other electrodes. The superior rate performance aligns with the high reversibility and stable voltage profiles observed during extended cycling, and can be attributed to the multiphase structure of NVOP, where conductive Ni_2_P facilitates electron transport while porous V-based oxide domains support fast lithium-ion diffusion.^68,69^ The corresponding voltage profiles of NVOP at different current densities (Figure 6e) exhibit moderate polarization and well-maintained curve shape, further confirming efficient charge-transfer kinetics and structural stability under increasing rate conditions.

To further evaluate the electrochemical kinetics of the electrodes, EIS measurements were carried out (Figure 6f). The equivalent circuit model and fitted parameters used for analysis are provided in the Supporting Information (Figure S8 and Table S3). NVOP electrode exhibits a significantly lower charge-transfer resistance (*R*_ct_) (1.96 Ω) compared to NVO (15.46 Ω) and NiV-LDH (3.55 Ω). This substantial reduction in *R*_ct_ highlights the superior interfacial charge-transfer kinetics of NVOP, which can be attributed to its unique architecture and high electrical conductivity. The low *R*_ct_ value reinforces the enhanced electrochemical activity and fast redox behavior of NVOP, corroborating its improved rate capability and cycling performance.

## 4 Conclusions

We present a new synthesis route for nickel vanadium oxyphosphide (NVOP) nanosheets, achieved through controlled phosphorization of NiV-LDH, and demonstrate their potential as high-performance anode materials for Li-ion batteries. Compared to their NiV-LDH and NVO counterparts, NVOP electrodes deliver significantly higher reversible capacities, superior rate capability, and excellent long-term cycling stability. Notably, after an initial phase of structural activation and capacity loss typical of conversion-type materials, NVOP rapidly stabilizes and retains a high capacity over extended cycling. This behavior is attributed to the synergistic interplay between conductive Ni_2_P, redox-active vanadium oxides, and a robust porous architecture, which together enable fast Li-ion transport, efficient charge transfer, and structural resilience. These results position NVOP as a compelling anode design that effectively addresses the instability challenges commonly associated with conversion reactions. Furthermore, the direct phosphorization strategy applied to a bimetallic layered double hydroxide precursor enables the formation of homogeneously distributed multiphase materials, combining the electrochemical advantages of each phase in a scalable and well-integrated structure.

## Supplementary Material



## Notes

Crystallographic and Rietveld refinement data (Tables S1 and S2); EDS mapping of NVOP, XPS spectra, BET/BJH analysis (Figures S1–S3); cyclic voltammetry (CV) of NiV-LDH and NVO (Figure S4), charge–discharge profiles (Figures S5); postcycling XRD (Figure S6); temperature-dependent cycling performances of NVOP (Figures S7); EIS fitting model and parameters (Figure S8, Table S3); and material comparison data (Table S4) (PDF)
