# Supplementary material for: Nickel Vanadium Oxyphosphide Nanosheets with Synergistic Metal–Phosphide Interfaces for Fast and Durable Lithium Storage
Source: ACS Appl Energy Mater. 2025 Sep 2;8(18):13451–61. doi: 10.1021/acsaem.5c01817 (PMC12458462; doi:10.1021/acsaem.5c01817)
Supplement: Supplementary file 1 [file ae5c01817_si_001.pdf]

Supporting Information

Nickel Vanadium Oxyphosphide Nanosheets with Synergistic Metal–Phosphide Interfaces for Fast and Durable Lithium Storage

Vivek Kumar Singh[1,2], *Idan Bar-lev*[1], *Keren Shwartsman*[1], *Srijith*[2], *Debabrata Mandal* [2], *Munseok S. Chae*[3], *Jeffrey D. Henderson*[4], *Mark C. Biesinger*[4,5], *Bibhudatta Malik*[2], *Gilbert Daniel Nessim*,[2]* *Daniel Sharon* [1]*

[1]*Department of Chemistry, Center for Nanoscience and Nanotechnology,*

Hebrew University of Jerusalem, Jerusalem, 91904, Israel

[2]*Department of Chemistry, Bar Ilan Institute for Nanotechnology and Advanced Materials, Bar Ilan University, Ramat Gan, 5290002, Israel*

[3]*Department of Nanotechnology Engineering, Pukyong National University, Busan 48547, Republic of Korea*

[4]*Surface Science Western, The University of Western Ontario, London, Ontario N6G 0J3, Canada*

[5]*Department of Chemistry, The University of Western Ontario, London, Ontario N6A 5B7, Canada*

* Corresponding Authors

Email: gdnessim@biu.ac.il, daniel.sharon@mail.huji.ac.il

Table S1. Crystallographic data and powder XRD Rietveld refinement results for NVO powder: atomic coordinates, site occupancies, isotropic displacement parameters, and reliability factors at room temperature.

Phase 1		NiO			
Crystal System		Cubic			
Space Group		F m -3 m			
Lattice Parameter		a = 4.075(1) Å,			
		V = 67.64(4) $Å^3$, Z = 4			
Atoms	x	y	z	Occupancy	U_{iso}
Ni(1)	0.0000	0.0000	0.0000	1.000	0.0032(1)
O(1)	0.0000	0.0000	0.5000	1.000	0.0032(1)
Phase 2		V_2O_3			
Crystal System		Trigonal			
Space Group		R -3 c			
Lattice Parameter		a = 5.163(6) Å, c = 15.41(3) Å,			
		V = 355.8(9) $Å^3$, Z = 6			
Atoms	x	y	z	Occupancy	U_{iso}
V(1)	0.0000	0.0000	0.1490(1)	1.000	0.0032(1)
O(1)	0.0000	0.3034(1)	0.2500	1.000	0.0032(1)

*R_p = 0.012, R_{wp} = 0.015, R_{exp} = 0.015, $R(F^2)$ = 0.086, χ^2 = 1.103

Table S2. Crystallographic data and powder XRD Rietveld refinement results for NVPO powder: atomic coordinates, site occupancies, isotropic displacement parameters, and reliability factors at room temperature.

Phase 1		NiO			
Crystal System		Cubic			
Space Group		F m -3 m			
Lattice Parameter		a = 4.223(4) Å,			
		V = 75.34(21) $Å^3$, Z = 4			
Atoms	x	y	z	Occupancy	U_{iso}
Ni(1)	0.0000	0.0000	0.0000	1.000	0.017(1)
O(1)	0.0000	0.0000	0.5000	1.000	0.017(1)
Phase 2		V_2O_3			
Crystal System		Trigonal			
Space Group		R -3 c			
Lattice Parameter		a = 5.080(10) Å, c = 14.45(5) Å,			
		V = 323.0(8) $Å^3$, Z = 6			

Atoms	x	y	z	Occupancy	U_{iso}
V(1)	0.0000	0.0000	0.1576(35)	1.000	0.017(1)
O(1)	0.0000	0.3121(35)	0.2500	1.000	0.017(1)
Phase 3		Ni_2P			
Crystal System		Hexagonal			
Space Group		P -6 2 m			
Lattice Parameter		a = 5.867(5) Å, c = 3.358(4) Å, V = 100.11(8) $Å^3$, Z = 3			
Atoms	x	y	z	Occupancy	U_{iso}
Ni(1)	0.0000	0.4048(10)	0.5000	1.000	0.043(1)
Ni(2)	0.0000	0.7460(10)	0.0000	1.000	0.043(1)
P(1)	0.3333	0.6667	0.0000	1.000	0.043(1)
P(2)	0.0000	0.0000	0.5000	1.000	0.043(1)

* R_p = 0.012, R_{wp} = 0.016, R_{exp} = 0.015, $R(F^2)$ = 0.221, χ^2 = 1.166

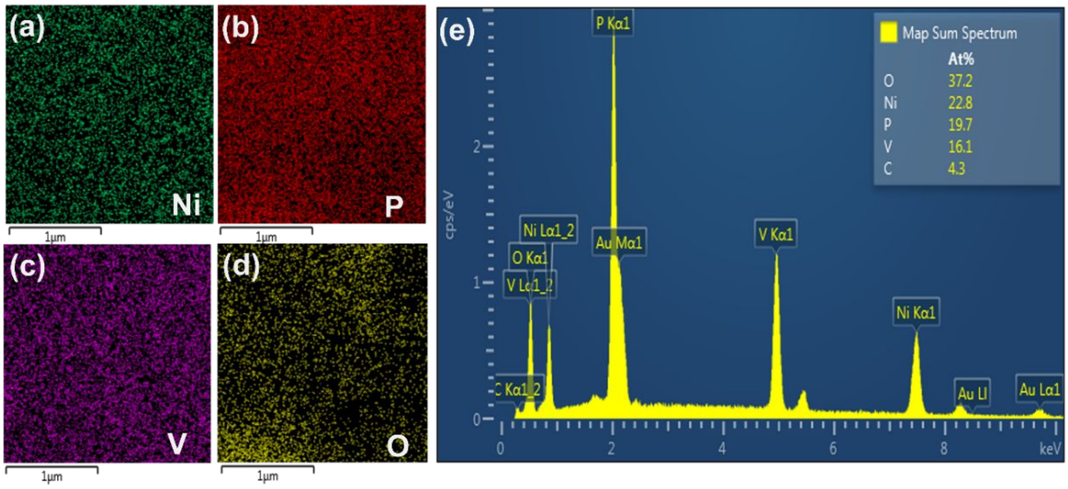

Figure S1.*(a-d) Energy-dispersive spectroscopy (EDS) elemental mapping showing uniform distribution of Ni (green), V (pink), P (red), and O (yellow), and (e) EDS spectra of NVOP.*

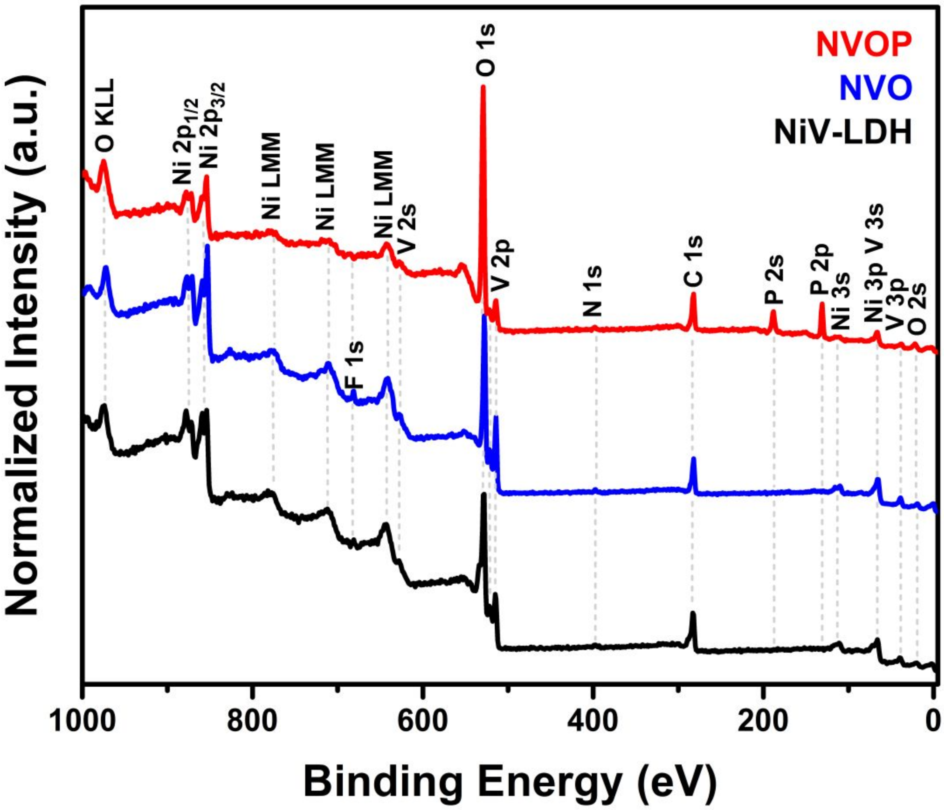

Figure S2. *Comparative XPS survey spectra of NiV-LDH, NVO, and NVOP.*

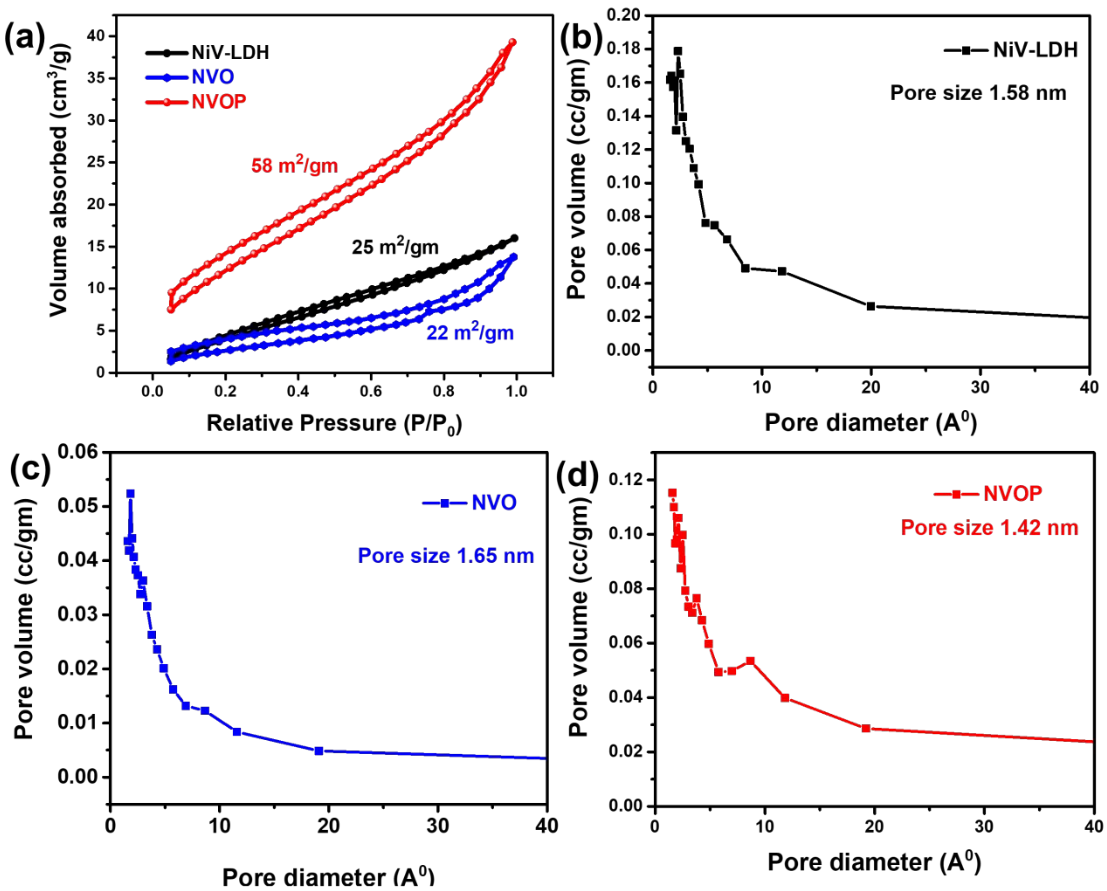

Figure S3. *(a) BET-N_2 adsorption/desorption isotherm of the NiV-LDH, NVO, and NVOP. (b-d) BJH desorption pore-size distribution of NiV-LDH, NVO, and NVOP.*

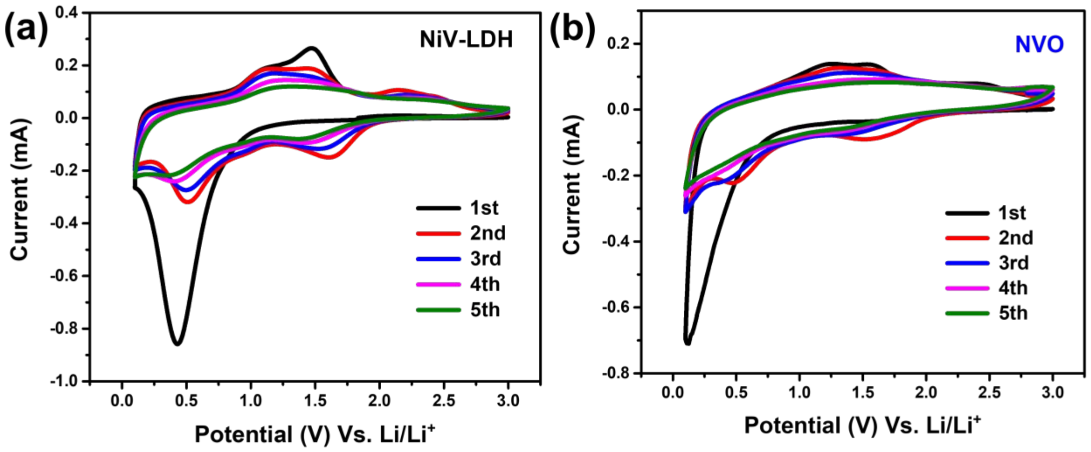

Figure S4. *CV curve of (a) NiV-LDH and (b) NVO at a scan rate of 0.1 mV/s from 0.1 to 3 V.*

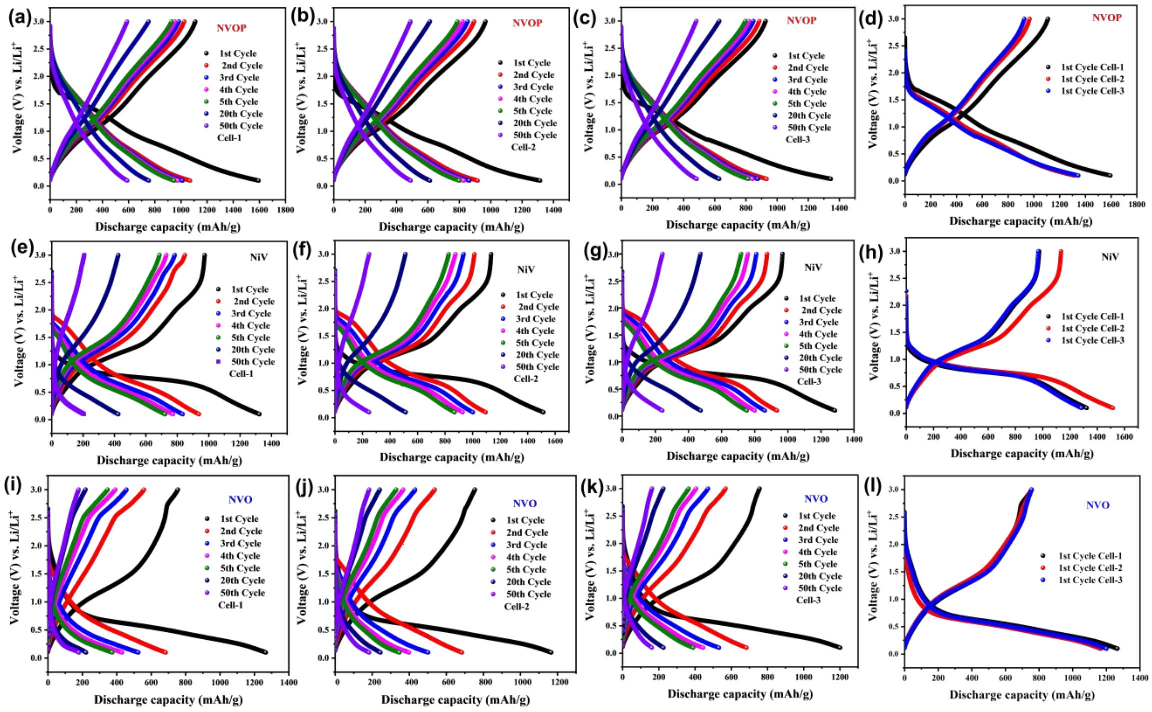

Figure S5. *Comparative galvanostatic charge−discharge curves performance of the three different cells of (a-d) NVOP, (e-h) NiV-LDH, and (i-l) NVO after the first five initial cycles, 20th and 50th cycles at a current density of 0.1 A/g.*

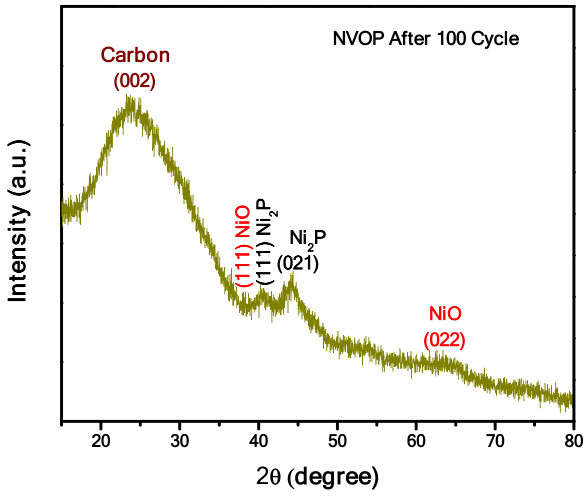

Figure S6. *X-ray diffraction pattern of the NVOP after 100 cycles.*

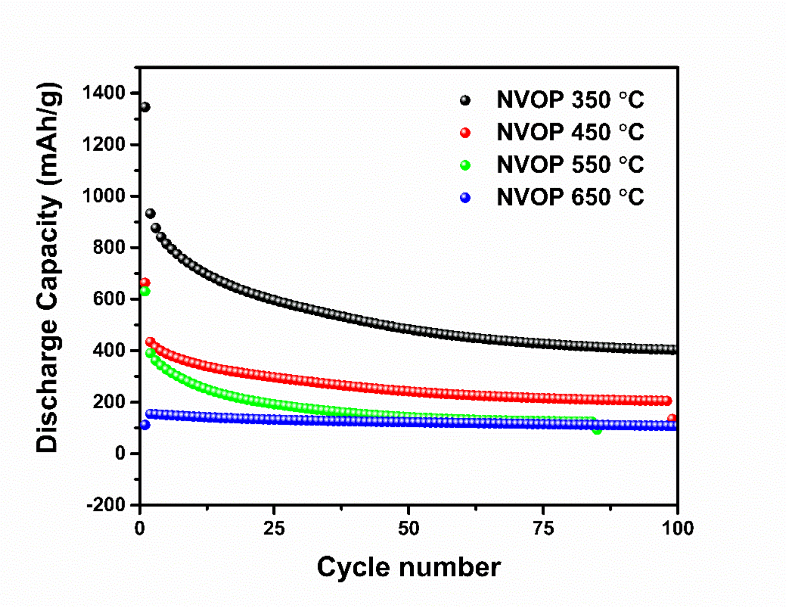

Figure S7. *Cycling performance of NVOP samples synthesized at different phosphorization temperatures (350 °C, 450 °C, 550 °C, and 650 °C) at a current density of 0.1 A g^{-1}.*

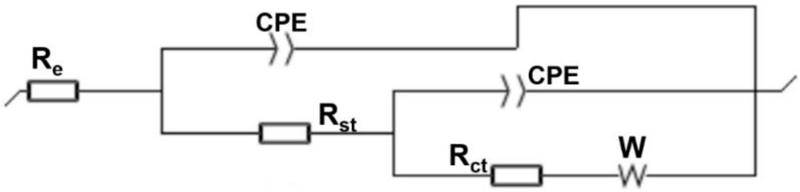

Figure S8. *Equivalent circuit model used to fit EIS data after the first cycle.*

Table S3. *Electrochemical kinetics parameters obtained from the equivalent circuit fitting of the EIS after the first cycle for the NiV-LDH, NVO, and NVOP*

Electrode	R_e (Ω)	R_{st} (Ω)	R_{ct} (Ω)
**NiV-LDH**	10.86	19.36	3.55
**NVO**	5.56	20.07	15.46
**NVOP**	6.917	15.33	1.96

Table S4. *Comparison of properties between NVOP and other Ni_2P-based materials*

S.N	Electrode	Capacity (mAh/g)	Cycle number	Current density (A/g)	Ref
1	**NVOP**	**442**	**200**	**0.1**	**This work**
2	Ni_2P@rGO	330.5	100	0.1	[1]
3	Ni_2P@NC	630	150	0.1	[2]
4	Ni_2P@C	610.4	400	0.1	[3]
5	Ni_2P nanosheet	379.8	50	0.1	[4]
6	Ni_2P/V_2O_5	440	200	0.1	[5]
7	Ni_2P Nanotubes	310	100	0.5	[6]
8	$FeP/Ni_2P/C$@C	426	100	0.5	[7]
9	Ni_2P/NF	507	100	0.05	[8]
10	Ni_5P_4/rGO	237.3	200	2	[9]
11	$Ni_2P/NG/Ni_2P$	417	100	0.3	[10]
12	$Ni_2P/NiS_{0.66}$	423.2	500	0.4	[11]

1. Cai, G.; Wu, Z.; Luo, T.; Zhong, Y.; Guo, X.; Zhang, Z.; Wang, X.; Zhong, B. 3D Hierarchical Rose-like Ni_2P@rGO Assembled from Interconnected Nanoflakes as Anode for Lithium-Ion Batteries. *RSC Adv.* **2020**, *10*, 3936–3945, doi:10.1039/C9RA10729K.
2. Fu, F.; He, Q.; Zhang, X.; Key, J.; Shen, P.; Zhu, J. Facile Synthesis of Nickel Phosphide @ N-Doped Carbon Nanorods with Exceptional Cycling Stability as Li-Ion and Na-Ion Battery Anode Material. *Batteries* **2023**, *9*, 267, doi:10.3390/batteries9050267.
3. Zhang, R.Z.; Zhu, K.J.; Huang, J.D.; Yang, L.Y.; Li, S.T.; Wang, Z.Y.; Xie, J.R.; Wang, H.; Liu, J. Ultrafine Ni_2P Nanoparticles Embedded in One-Dimensional Carbon Skeleton Derived from Metal-Organic Frameworks Template as a High-Performance Anode for Lithium Ion Battery. *J. Alloys Compd.* **2019**, *775*, 490–497, doi:10.1016/j.jallcom.2018.10.058.
4. Lu, Y.; Tu, J.; Xiong, Q.; Zhang, H.; Gu, C.; Wang, X.; Mao, S.X. Large-Scale Synthesis of Porous Ni_2P Nanosheets for Lithium Secondary Batteries. *CrystEngComm* **2012**, *14*, 8633, doi:10.1039/c2ce26378e.
5. Yu, Y.; Huang, S.; Wang, B.; Tie, D.; Wang, Q.; Hou, Y.; Zhao, Y. Achieving High-Energy Full-Cell Lithium-Storage Performance by Coupling High-Capacity V_2O_3 with

Low-Potential Ni_2P Anode. *ACS Appl. Mater. Interfaces* **2019**, *11*, 19–25, doi:10.1021/acsami.8b17910.
6. Lu, Y.; Tu, J.; Xiong, Q.; Qiao, Y.; Zhang, J.; Gu, C.; Wang, X.; Mao, S.X. Carbon-Decorated Single-Crystalline Ni_2P Nanotubes Derived from Ni Nanowire Templates: A High-Performance Material for Li-Ion Batteries. *Chem. Eur. J.* **2012**, *18*, 6031–6038, doi:10.1002/chem.201103724.
7. Yao, C.; Zha, J.; Li, C.; Wang, Z.; Shen, Y.; Xie, A. Yolk-Shelled FeP/Ni_2P/C@C Nanospheres with Void: Controllable Synthesis and Excellent Performance as the Anode for Lithium-Ion Batteries. *Colloids Surf. A.* **2020**, *602*, 125103, doi:10.1016/j.colsurfa.2020.125103.
8. Li, Q.; Ma, J.; Wang, H.; Yang, X.; Yuan, R.; Chai, Y. Interconnected Ni_2P Nanorods Grown on Nickel Foam for Binder Free Lithium-Ion Batteries. *Electrochim. Acta.* **2016**, *213*, 201–206, doi:10.1016/j.electacta.2016.07.105.
9. Guo, H.; Chen, C.; Chen, K.; Cai, H.; Chang, X.; Liu, S.; Li, W.; Wang, Y.; Wang, C. High Performance Carbon-Coated Hollow $Ni_{12}P_5$ Nanocrystals Decorated on GNS as Advanced Anodes for Lithium and Sodium Storage. *J. Mater. Chem. A* **2017**, *5*, 22316–22324, doi:10.1039/C7TA06843C.
10. Dong, C.; Guo, L.; He, Y.; Chen, C.; Qian, Y.; Chen, Y.; Xu, L. Sandwich-like Ni_2P Nanoarray/Nitrogen-Doped Graphene Nanoarchitecture as a High-Performance Anode for Sodium and Lithium-Ion Batteries. *Energy Storage Mater.* **2018**, *15*, 234–241, doi:10.1016/j.ensm.2018.04.011.
11. Wu, T.; Zhang, S.; He, Q.; Hong, X.; Wang, F.; Wu, X.; Yang, J.; Wen, Z. Assembly of Multifunctional $Ni_2P/NiS_{0.66}$ Heterostructures and Their Superstructure for High Lithium and Sodium Anodic Performance. *ACS Appl. Mater. Interfaces* **2017**, *9*, 28549–28557, doi:10.1021/acsami.7b07939.
